# Impact of umbilical cord milking and pasteurized donor human milk on necrotizing enterocolitis: a retrospective review

**DOI:** 10.1186/s12887-018-1131-x

**Published:** 2018-05-08

**Authors:** Mehtab K. Sekhon, Bradley A. Yoder

**Affiliations:** Division of Neonatology, University of Utah School of Medicine, 295 Chipeta Way, Salt Lake City, UT 84108 USA

**Keywords:** Pasteurized donor human milk, Necrotizing enterocolitis, Umbilical cord milking

## Abstract

**Background:**

Necrotizing enterocolitis (NEC) is a serious complication of prematurity. Our objective was to evaluate the impact of an umbilical cord milking protocol (UCM) and pasteurized donor human milk (PDHM) on NEC rates in infants less than 30 weeks gestational age from January 1, 2010 to September 30, 2016. We hypothesized an incremental decrease in NEC after each intervention.

**Methods:**

We performed a retrospective review of 638 infants born less than 30 weeks gestational age. Infants were grouped into three epochs: pre-UCM/pre-PDHM (Epoch 1, *n* = 159), post-UCM/pre-PDHM (Epoch 2, *n* = 133), and post-UCM/post-PDHM (Epoch 3, *n* = 252). The incidence of NEC, surgical NEC, and NEC/death were compared. Logistic regression was used to determine independent significance of time epoch, gestational age, birth weight, and patent ductus arteriosus for NEC, surgical NEC, and death/NEC.

**Results:**

At birth, infants in Epoch 1 were younger than Epoch 2 and 3 (26.8 weeks versus 27.3 and 27.2, respectively, *P* = 0.036) and smaller (910 g versus 1012 and 983, respectively, *P* = 0.012). Across epochs, there was a significant correlation between patent ductus arteriosus treatment and NEC rate (*P* < 0.001, Cochran-Mantel-Haenszel). There was a significant decrease in rates of NEC, surgical NEC, and NEC/death between groups. Logistic regression showed this as significant for rates of NEC and surgical NEC between Epoch 1 and 3. Patent ductus arteriosus was a significant variable affecting the incidence of NEC, but not surgical NEC or death/NEC.

**Conclusions:**

An umbilical cord milking protocol and pasteurized donor human milk availability was associated with decreased rates of NEC and surgical NEC. This suggests an additive effect of these interventions in preventing NEC.

## Background

Necrotizing enterocolitis (NEC) is a serious complication of prematurity, with a prevalence of 7% in very low birth weight (VLBW) infants and mortality between 20 and 30% [1]. Preventative strategies include small volume enteral feeds (feeding advances of 10-20 ml/kg/day), human milk, and probiotics [1]. Pasteurized donor human milk (PDHM) is an alternative to mother’s own milk when a mother’s supply is inadequate [2]. Retrospective and prospective randomized trials have reported reduced rates of NEC with donor milk versus formula, when maternal breast milk was unavailable [3, 4]. However, not all trials have shown differences in NEC rates [2, 5].

Umbilical cord milking (UCM) has been reported to decrease the risk of developing NEC [6, 7]. UCM, one approach to placental transfusion, results in the active transfer of fetal placental blood to the infant over 15–30 s [7–9]. It has been associated with increased blood volumes, reduced red blood cell transfusions, higher mean blood pressures, and less inotrope therapy in very preterm infants, presumably promoting end-organ perfusion [6, 9–12].

What is unknown is the cumulative impact of PDHM and UCM on rates of NEC. The University of Utah Neonatal Intensive Care Unit (NICU) instituted two process changes over a seven-year period: an UCM protocol in September 2011 and providing PDHM to infants of mothers unable to provide their own milk starting June 2013. The UCM protocol sought to reduce the combined outcome of severe intraventricular hemorrhage, NEC, and/or death prior to discharge. The results of this process change were published by Patel et al. (2014). Since both UCM and PDHM can reduce NEC but were introduced at different times, we investigated whether there was an effect on the rate of NEC associated with each process. We hypothesized an incremental decrease in the incidence of NEC following each intervention.

## Methods

Eligible infants were identified from a database of neonates less than 30^0/7^ weeks gestational age admitted to the University of Utah NICU between January 1, 2010 and September 30, 2016. This database contains prospectively captured morbidity and mortality information for each neonate, including whether an infant developed NEC and if so, the stage (Bell stage ≥2 or surgical NEC [13]). The data is not reported externally and is available to researchers within our institution. Eligibility criteria included gestational age at birth from 23^0/7^ to 29^6/7^ weeks and inborn between the study period. Infants with lethal or severe congenital anomalies were excluded. Infants were categorized into three time epochs: pre-umbilical cord milking and pre-donor human milk (Epoch 1); post-umbilical cord milking and pre-donor human milk (Epoch 2); post-umbilical cord milking and post-donor human milk (Epoch 3). Institutional review board approval and a waiver of informed consent was obtained.

The UCM protocol was adapted from Hosono et al. (2008) and applied to infants born less than 30 weeks gestational age. Once delivered, the infant was held 10 cm below the level of the placenta. The umbilical cord was pinched close to the placenta and milked towards the infant over 2–3 s. This was repeated three times over a total period of less than 30 s. The cord was clamped and the infant handed to the neonatal team for resuscitation. No resuscitation took place during cord milking.

Infants less than 1800 g or born at less than 34 weeks gestational age qualify for PDHM. Our unit did not have a standardized feeding protocol during the study period. However, trophic feeds were typically started within the first 24-48 h of life and full feeds reached by 14 days of life. Enteral feeds are fortified with a bovine based human milk fortifier. No new nutrition protocols were introduced during the study period. Infants qualifying for PDHM in Epoch 3 could receive mother’s own milk only, PDHM only, or a combination of both. Infants in Epoch’s 1 and 2 received mother’s own milk and/or formula.

The primary outcome was the incidence of any NEC between epochs. Secondary outcomes included differences in rates of surgical NEC, any cause death, and death secondary to NEC between epochs. Spontaneous intestinal perforation was differentiated from surgical NEC based on clinical presentation and operative findings [14].

The primary outcome and other categorical measures were analyzed by Chi-square or Fisher’s Exact test. Student’s *t* test was used for analysis of normally distributed continuous data and Mann-Whitney *U* test was applied for ordinal data or continuous data that was not normally distributed. Two-sided *P* values less than 0.05 were statistically significant and no adjustment was made for multiple comparisons. A panel of risk factors associated with NEC or surgical NEC was developed from initial unadjusted analyses, using a *P* value < 0.10 for inclusion. Logistic regression analysis was applied to determine independent significance of identifiable risk factors, including time epoch, for NEC, surgical NEC, and death or NEC. Risk factors were determined to be significant if the 95% confidence intervals did not cross 1. Statistical analysis was performed using SPSS (version 24, IBM, Armonk NY).

## Results

**Fig. 1:**
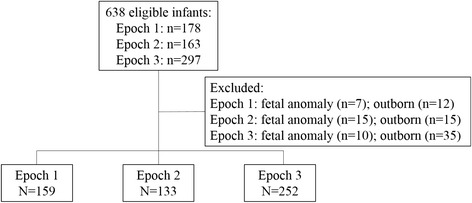
Patient flowchart depicting inclusions and exclusions in each epoch

**Fig. 2:**
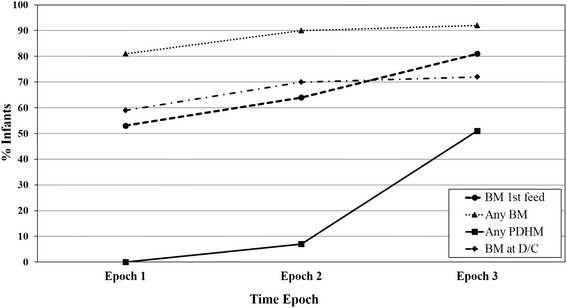
Sequential changes in breast milk (BM) use across time epochs are shown. Any BM use, BM at first feed and BM at discharge (D/C) gradually increased over time. There was a dramatic increase in the use of pasteurized donor human milk (PDHM) in lieu of formula in Epoch 3. Raw data was not available to allow statistical comparisons

Figure 1 shows the patient flowchart. Amongst 638 infants, 94 were excluded for a total study group of 544. Cord milking was not performed in Epoch 1. Subsequently, UCM was performed in 114/133 (86%) of subjects in Epoch 2 and 238/252 (94%) subjects in Epoch 3. PDHM became available in June 2013; the beginning of Epoch 3. As seen in Fig. 2, during Epoch 3, there was an increase in PDHM use, in lieu of formula, with over 50% of infants receiving at least some PDHM. The majority of PDHM use was in the initial days of life, with first feeding as human milk increasing from 53% in Epoch 1 (mother’s own milk only) to 81% in Epoch 3 (mother’s own milk and/or PDHM). Additionally, the age of initial feeding decreased from a mean of 2.9 to 1.2 days of age. Availability of mother’s own milk increased during Epoch 1, from 82 to 91%, and remained stable between 90 and 91% during Epoch 2. Data on rates of mother’s own milk usage only are not available for Epoch 3. Rates of any formula use declined during Epoch 1 from 19 to 9%, and remained stable at 9% during Epoch’s 2 and 3. Patient characteristics are shown in Table 1. Infants in Epoch 1 were significantly younger and smaller at birth. Over the three epochs, there was a shift in maternal race/ethnicity, with increasing Hispanic representation; decreased diagnosis of chorioamnionitis and maternal intrapartum antibiotic use; and increased use of intrapartum magnesium sulfate prophylaxis (Table 1). Cochran-Mantel-Haenszel test did not show a significant relationship between these changes in maternal characteristics and NEC rates across epochs. We also noted a decreasing rate of patent ductus arteriosus (PDA) treatment across epochs, with a significant decrease in PDA ligation noted in Epoch 3 (Table 1). There was a significant association between PDA treatment and NEC rate across epochs (Cochran-Mantel-Haenszel test, *P* < 0.001).

**Table 1:** Patient demographics for each time epoch

	Epoch 1 *n* = 159	Epoch 2 *n* = 133	Epoch 3 *n* = 252	P
Gestational age (SD)	26.8 (1.9)	27.3 (1.9)	27.2 (1.9)	0.036
23–24 weeks	38 (24%)	14 (11%)	47 (19%)	
25–26 weeks	39 (25%)	43 (32%)	55 (22%)	
27–29 weeks	82 (52%)	76 (57%)	150 (60%)	
Birth weight (SD)	911 (304)	1011 (306)	983 (300)	0.012
≤ 750 g	56 (35%)	25 (19%)	63 (25%)	
751-999 g	42 (62%)	47 (35%)	71 (28%)	
≥ 1000 g	61 (38%)	61 (46%)	118 (47%)	
Sex - Male (n,%)	84 (53%)	75 (56%)	131 (52%)	0.705
SGA	16 (10%)	18 (14%)	33 (13%)	0.585
Maternal factors				
Race				0.005
Caucasian	105 (66%)	85 (64%)	161 (64%)	
Black	10 (6%)	3 (2%)	5 (2%)	
Hispanic	24 (15%)	34 (26%)	71 (28%)	
Other	20 (13%)	11 (8%)	15 (6%)	
Antenatal steroids - any	154 (97%)	130 (98%)	244 (97%)	0.865
Chorioamnionitis - clinical	48 (30%)	27 (20%)	34 (13%)	< 0.001
Antibiotics – peripartum	115 (72%)	96 (72%)	125 (50%)	< 0.001
Intrapartum Mg sulfate	108 (68%)	100 (75%)	228 (90%)	< 0.001
C-section delivery	110 (69%)	87 (65%)	189 (75%)	0.121
Apgar Median (25–75%)				
1 min	5 (2–7)	4 (2–7)	4 (2–7)	0.780
5 min	7 (6–8)	7 (6–8)	7 (6–8)	0.955
PDA treatment (n,%)	57 (36%)	40 (30%)	65 (26%)	0.094
Medical Only	36 (63%)	23 (58%)	51 (78%)	0.526
Surgical Ligation Only	11 (19%)	6 (15%)	1 (2%)	0.001
Medical & Surgical ligation	10 (18%)	11 (28%)	13 (20%)	0.487

Data as mean (SD) or number (%), Epoch 1 pre-umbilical cord milking and pre-donor human milk; Epoch 2 post-umbilical cord milking and pre-donor human milk, Epoch 3 post-umbilical cord milking and post-donor human milk; *SGA* small for gestational age, *Mg* magnesium; *PDA* patent ductus arteriosus

**Fig. 3:**
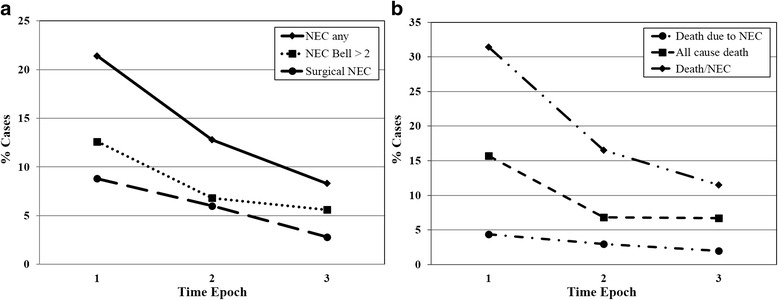
**a**. Sequential change in rate of NEC (*p* = 0.002) and surgical NEC (*p* = 0.002), between Epochs 1, 2, and 3. **b**. Changes in all cause death (*p* = 0.003), death due to NEC (*p* = 0.070), and combined outcome of death/NEC (*p* < 0.001). NEC – necrotizing enterocolitis; Epoch 1 – pre-umbilical cord milking and pre-donor human milk; Epoch 2 – post-umbilical cord milking and post-donor human milk; Epoch 3 – post-umbilical cord milking and pre-donor human milk

There were 35 (20%) cases of any NEC in Epoch 1, 20 (12%) in Epoch 2, and 28 (9%) in Epoch 3. There was a significant decrease in rates of NEC (*p* = 0.002), surgical NEC (*p* = 0.002), all cause death (*p* = 0.003), and death or NEC (*p* = < 0.001) across epochs (Fig. 3). Using Epoch 1 as a reference, logistic regression analysis showed significant reduction in the rates of NEC, surgical NEC, and death or NEC between Epoch 3 and 1 (Table 2). There was also a significant reduction in death or NEC noted for Epoch 2 versus 1. PDA requiring treatment was a significant variable affecting the rate of NEC, but not surgical NEC or death or NEC. Gestational age was a significant factor affecting the combined outcome of NEC or death.

**Table 2:** Logistic regression analysis of impact of time epoch, gestational age, birth weight, and treated PDA on NEC rates, surgical NEC, and NEC or Death

Data as OR (95% CI)	NEC cases	Surgical NEC	Death or NEC
Time epoch (Epoch 1 = Ref)			
Epoch 2	0.58 (0.30–1.12)	0.87 (0.34–2.23)	0.51 (0.28–0.93)
Epoch 3	0.37 (0.20–0.67)	0.36 (0.14–0.92)	0.30 (0.18–0.52)
Gestational age, week	0.91 (0.74–1.12)	0.77 (0.57–1.05)	0.75 (0.63–0.91)
Birth weight, gram	1.00 (0.99–1.00)	0.99 (0.99–1.00)	0.99 (0.99–1.00)
PDA treated	2.17 (1.25–3.76)	1.23 (0.55–2.74)	0.83 (0.50–1.38)

*PDA* patent ductus arteriosus, *NEC* necrotizing enterocolitis, *OR* odds ratio, *CI* confidence interval; Epoch 1 pre-umbilical cord milking and pre-donor human milk, Epoch 2 post-umbilical cord milking and post-donor human milk, Epoch 3 post-umbilical cord milking and pre-donor human milk

## Discussion

This retrospective review of the effect of an UCM protocol followed by PDHM availability on NEC rates showed an incremental decrease in NEC after each process change. We observed significant decreases in rates of surgical NEC, all cause death, and death or NEC. By logistic regression analysis, this association was significant for rates of NEC, surgical NEC, and death or NEC between Epochs 1 and 3. This suggests that the combined effects of the UCM protocol and PDHM availability were associated with decreased rates of NEC and surgical NEC.

Multiple reviews have identified lower rates of NEC among infants undergoing UCM or delayed cord clamping versus immediate cord clamping [6, 15, 16]. Dang et al’s review looked at UCM versus immediate cord clamping and found a 40 and 50% decreased NEC risk and all cause death, respectively, in the UCM group. Similarly, Rabe et al’s (2012) review reported a 38% reduction in NEC risk in infants undergoing UCM or delayed cord clamping. Thus, these studies, in conjunction with our findings, provide further support for UCM, particularly in VLBW infants. In contrast, no differences in NEC were seen in a study assessing the effect of UCM versus immediate cord clamping on cerebral and systemic perfusion in VLBW neonates [10]. Additionally, March et al’s [17] study found no difference in NEC or all cause death in infants undergoing UCM versus immediate cord clamping. This study may have been underpowered to detect NEC, as it was a secondary outcome, and did not report baseline NEC rates, which would influence the significance of any decreases in NEC.

Similar to our findings, Chowning et al. [3] reported a reduction in any NEC and surgical NEC in VLBW infants following introduction of PDHM. These findings were confirmed in the randomized trial of O’Connor et al. [4], with significantly lower NEC rates in PDHM versus formula groups. Finally, Cristofalo et al. [5] found significant decreases in rates of surgical NEC in infants receiving PDHM versus formula. However, the decrease in any NEC was not statistically significant, perhaps due to population and center differences, as their study was multicenter and included large metropolitan centers, while ours is a single center study with a homogenous population. Also, their study involved exclusive PDHM use, whereas subjects in our study received a mix of mother’s milk and/or PDHM.

PDA treatment was an associated risk factor for any diagnosis of NEC across epochs. Previous studies have suggested an association between PDA and NEC [18]. However, neither medical nor surgical treatment prevent NEC in very low birth weight infants [19, 20]. Thus, although an associated finding, it seems unlikely the decrease in PDA treatment was clinically related to decreased NEC over time.

The pathophysiology of NEC involves an abnormal intestinal microbiome and immaturity of the intestinal barrier, microvasculature, and immune system [1, 21]. Placental transfusion, through UCM or delayed cord clamping, improves circulating blood volume and is accompanied by higher mean blood pressures [6, 9, 11, 12]. Thus, UCM may prevent or minimize early intestinal ischemic injury, protecting against NEC. PDHM may protect against NEC by providing factors that modulate the immune system, promote development of the intestinal mucosa, and facilitate growth of a favorable intestinal microbiome [21–23]. While Holder pasteurization of donor milk results in the loss of some of these factors, many are still preserved or only mildly reduced [22]. Furthermore, PDHM is bactericidal, despite pasteurization [22].

There are several limitations to our study. First, logistic regression showed significant differences in rates of NEC, surgical NEC, and death/NEC between Epoch’s 1 and 3, but not 1 and 2. However, we observed decreases in rates of NEC and surgical NEC across both Epoch 2 and 3. The lack of statistical significance may be due to limited sample size and power, given 133 patients in Epoch 2 versus 252 in Epoch 3. Also, PDHM may have had a greater effect on NEC rates than UCM, though a greater relative decrease in NEC was noted between Epoch 1 and 2, than between Epoch’s 2 and 3. Additionally, generalizability may be limited, as this is a retrospective study involving a single center serving a predominantly Caucasian population. There was a significant difference in gestational age and birth weight between subjects in each epoch, with more infants between 23 and 24 weeks gestation and ≤ 750 g in Epoch 1. However, regression modeling did not show gestational age or birth weight as significant independent factors affecting NEC rates. Rates of mother’s own milk use may have been lower in Epoch 1 due to increased maternal stress [24], as infants were younger and smaller, and potentially sicker, and more mothers had clinical chorioamnionitis. In addition, a greater percentage of Hispanic mothers in Epoch’s 2 and 3 may have increased rates of mother’s own milk use, as Hispanic mothers have higher rates of breastfeeding [25]. However, use of mother’s own milk increased in Epoch 1 and overall rates in Epoch’s 1 and 2 were higher than the average rates of mother’s own milk use in NICU’s across the United States [26]. Although we do not have mother’s own milk use data for Epoch 3, there is no reason to suspect a decline in availability of mother’s own milk during this epoch. No other major care process changes were introduced during Epoch 2 and 3. Nonetheless, other general improvements in clinical practice could contribute to the observed reductions in NEC from 2010 to 2016. For example, we showed a decrease in intrapartum antibiotics and increase in magnesium sulfate for neuroprophylaxis. We are unaware of any association with intrapartum antibiotic use and subsequent NEC, though prolonged initial antibiotic therapy of the premature neonate has been associated with increased NEC risk [27]. We do not have data on initial antibiotic use in this study group. Recent reports have suggested an association of spontaneous intestinal perforation, and possibly NEC, among extremely preterm infants less than 26 weeks gestation exposed to antenatal magnesium prophylaxis [28, 29]. Our study excluded spontaneous intestinal perforation, which has a different pathophysiology than NEC [14]. If NEC rates increase with increased exposure to intrapartum magnesium sulfate for neuroprophylaxis, we should expect an increase in NEC rates rather than the decrease observed. Finally, we did not account for other risk factors for NEC, such as antibiotic exposure, postnatal steroid use, packed red blood cell transfusions, and sepsis.

Our study’s strengths include a prospectively maintained database of a high risk population targeting major neonatal morbidities and a large sample size across epochs. Although a single center analysis, our unit is a level III NICU in an academic center serving a large urban population and receives neonatal and maternal transfers from across Utah and neighboring states. Additionally, our NEC rates are within the range of similar sized academic centers within the neonatal research network, as reported by Stoll et al. [30]. In this report, the average NEC rate in infants born between 22 and 29 weeks gestational age was 11% (4–19%), with higher rates observed when infants were sub-categorized by gestational age. Furthermore, we had excellent compliance with UCM, with 86 and 94% of infants receiving UCM in Epoch 2 and 3, respectively. This further demonstrates the ease of provider adherence to UCM. Moreover, during Epoch 3, at least half of infants qualifying for PDHM received it and there were increased rates of first feeds as human milk. All infants were inborn, negating variability in initial care between referring institutions.

## Conclusions

In summary, we identified incremental decreases in rates of NEC and surgical NEC associated with implementation of an UCM protocol followed by a protocol for providing PDHM to infants of mothers unable to provide their own milk. This suggests an additive effect of these interventions in preventing NEC. Whether these interventions are additive or potentially synergistic cannot be determined from this study.

## Abbreviations

Epoch 1: Pre-umbilical cord milking and pre-donor human milk
Epoch 2: Post-umbilical cord milking and pre-donor human milk
Epoch 3: Post-umbilical cord milking and post-donor human milk
GA: Gestational age
NEC: Necrotizing enterocolitis
NICU: Neonatal intensive care unit
PDA: Patent ductus arteriosus
PDHM: Pasteurized donor human milk
UCM: Umbilical cord milking
VLBW: Very low birth weight

## References

[1] Neu J, Walker WA (2011). Necrotizing enterocolitis. N Engl J Med.

[2] Schanler RJ, Lau C, Hurst NM, Smith EO (2005). Randomized trial of donor human milk versus preterm formula as substitutes for mothers own milk in the feeding of extremely premature infants. Pediatrics.

[3] Chowning R, Radmacher P, Lewis S, Serke L, Pettit N, Adamkin DH (2016). A retrospective analysis of the effect of human milk on prevention of necrotizing enterocolitis and postnatal growth. J Perinatol.

[4] O’Connor DL, Gibbins S, Kiss A, Bando N, Brennan-Donnan J, Ng E, Campbell DM, Vaz S, Fusch C, Asztalos E, Church P, Kelly E, Ly L, Daneman A, Unger S (2016). Effect of supplemental donor human milk compared with preterm formula on neurodevelopment of very low-birth-weight infants at 18 months: a randomized clinical trial. JAMA.

[5] Cristofalo EA, Schanler RJ, Blanco CL, Sullivan S, Trawoeger R, Kiechl-Kohlendorfer U, Dudell G, Rechtman DJ, Lee ML, Lucas A, Abrams S (2013). Randomized trial of exclusive human milk versus preterm formula diets in extremely premature infants. J Pediatr.

[6] Dang D, Zhang C, Shi S, Mu X, Lv X, Wu H (2015). Umbilical cord milking reduces need for red cell transfusions and improves neonatal adaptation in preterm infants: meta-analysis. J Obstet Gynaecol Res.

[7] Al-Wassia H, Shah PS (2015). Efficacy and safety of umbilical cord milking at birth: a systematic review and meta-analysis. JAMA Pediatr.

[8] Hosono S, Hine K, Nagano N, Taguchi Y, Yoshikawa K, Okada T, Mugishima H, Takahashi S, Takahashi S (2015). Residual blood volume in the umbilical cord of extremely premature infants. Pediatr Int.

[9] Hosono S, Mugishima H, Fujita H, Hosono A, Minato M, Okada T, Takahashi S, Harada K (2008). Umbilical cord milking reduces the need for red cell transfusions and improves neonatal adaptation in infants born at less than 29 weeks gestation: a randomized controlled trial. Arch Dis Child Fetal Neonatal Ed.

[10] Takami T, Suganami Y, Sunohara D, Kondo A, Mizukaki N, Fujioka T, Hoshika A, Akutagawa O, Isaka K (2012). Umbilical cord milking stabilizes cerebral oxygenation and perfusion in infants born before 29 weeks of gestation. J Pediatr.

[11] Katheria AC, Truong G, Cousins L, Oshiro B, Finer NN (2015). Umbilical cord milking versus delayed cord clamping in preterm infants. Pediatrics.

[12] Patel S, Clark EAS, Rodriguez C, Metz TD, Abbaszadeh M, Yoder BA (2014). Effect of umbilical cord milking on morbidity and survival in extremely low gestational age neonates. Am J Obstet Gynecol.

[13] Kliegman RM, Walsh MC (1987). Neonatal necrotizing enterocolitis: pathogenesis, classification, and spectrum of illness. Curr Probl Pediatr.

[14] Pumberger W, Mayr M, Kohlhauser C, Weninger M (2002). Spontaneous localized intestinal perforation in very-low-birth-weight infants: a distinct clinical entity different from necrotizing enterocolitis. J Am Coll Surg.

[15] Rabe H, Diaz-Rossello JL, Duley L, Dowswell T. Effect of timing of umbilical cord clamping and other strategies to influence placental transfusion at preterm birth on maternal and infant outcomes. Cochrane Database Syst Rev. 2012. 10.1002/14651858.CD003248.pub3. 10.1002/14651858.CD003248.pub3 22895933

[16] Safarulla A (2017). A review of benefits of cord milking over delayed cord clamping in the preterm infant and future directions of research. J Matern Fetal Neonatal Med.

[17] March MI, Hacker MR, Parson AW, Modest AM, de Veciana M (2013). The effects of umbilical cord milking in extremely preterm infants: a randomized controlled trial. J Perinatol.

[18] Dollberg S, Lusky A, Reichman B (2005). Patent ductus arteriosus, indomethacin and necrotizing enterocolitis in very low birth weight infants: a population-based study. J Pediatr Gastroenterol Nutr.

[19] Yee WH, Scotland J (2012). Evidence-based Practice for Improving Quality (EPIQ) Evidence Review Group. Does primary surgical closure of the patent ductus arteriosus in infants <1500 g or ≤32 weeks’ gestation reduce the incidence of necrotizing enterocolitis?. Paediatr Child Health.

[20] Sung SI, Chang YS, Chun JY, Yoon SA, Yoo HS, Ahn SY, Park WS (2016). Mandatory closure versus nonintervention for patent ductus arteriosus in very preterm infants. J Pediatr.

[21] Nino DF, Sodhi CP, Hackam DJ (2016). Necrotizing enterocolitis: new insights into pathogenesis and mechanisms. Nat Rev Gastroenterol Hepatol.

[22] Arslanoglu S, Corpeleijn W, Moro G, Braegger C, Campoy C, Colomb V, Decsi T, Domellof M, Fewtrell M, Hojsak I, Mihatsch W, Molgaard C, Shamir R, Turck D, van Goudoever J (2013). Donor human milk for preterm infants: current evidence and research directions. J Pediatr Gastroenterol Nutr.

[23] Lewis ED, Richard C, Larsen BM, Field CJ (2017). The importance of human milk for immunity in preterm infants. Clin Perinatol.

[24] Hill PD, Aldag JC, Chatterton RT, Zinaman M (2005). Psychological distress and milk volume in lactating mothers. West J Nurs Res.

[25] McKinney CO, Hahn-Holbrook J, Chase-Lansdale L, Ramey SL, Krohn J, Reed-Vance M, Raju TNK, Shalowitz MU (2016). Racial and ethnic differences in breastfeeding. Pediatrics.

[26] Boundy EO, Perrine CG, Nelson JM, Hamner HC (2017). Disparities in hospital-reported breast milk use in neonatal intensive care units – United States, 2015. MMWR Morb Mortal Wkly Rep.

[27] Cotten CM, Taylor S, Stoll B, Goldberg RN, Hansen NI, Sánchez PJ, Ambalavanan N, Benjamin DK (2009). NICHD Neonatal Research Network. Prolonged duration of initial empirical antibiotic treatment is associated with increased rates of necrotizing enterocolitis and death for extremely low birth weight infants. Pediatrics.

[28] Rattray BN, Kraus DM, Drinker LR, Goldberg RN, Tanaka DT, Cotten CM (2014). Antenatal magnesium sulfate and spontaneous intestinal perforation in infants less than 25 weeks gestation. J Perinatol.

[29] Kamyar M, Clark EA, Yoder BA, Varner MW, Manuck TA (2016). Antenatal magnesium sulfate, necrotizing enterocolitis, and death among neonates < 28 weeks gestation. AJP Rep.

[30] Stoll BJ, Hansen NI, Bell EF, Shankaran S, Laptook AR, Walsh MC, Hale EC, Newman NS, Schibler K, Carlo WA, Kennedy KA, Poindexter BB, Finer NN, Ehrenkranz RA, Duara S, Sanchez PJ, O’Shea TM, Goldberg RN, Van Meurs KP, Faix RG, Phelps DL, Frantz ID, Watterberg KL, Saha S, Das A, Higgins RD (2010). Neonatal outcomes of extremely preterm infants from the NICHD neonatal research network. Pediatrics.

